# Surgical Technique and Oro-Nasal Fistula Formation After Primary Palatoplasty: A Comparative Study of Closed Intravelar and Modified Veau–Wardill–Kilner Techniques

**DOI:** 10.3390/jcm15082825

**Published:** 2026-04-08

**Authors:** Kostadin Gigov, Ivan Ginev, Petra Kavradjieva, Ivaylo Minev, Mariya Miteva

**Affiliations:** 1 Section of Plastic Reconstructive and Aesthetic Surgery and Thermal Trauma, Department of Propedeutics of Surgical Diseases, Faculty of Medicine, “St. George” University Hospital Plovdiv, Medical University of Plovdiv, “Peshtersko shausse Blvd 66”, 4002 Plovdiv, Bulgaria; kostadin.gigov@phd.mu-plovdiv.bg (K.G.); ivan.ginev@mu-plovdiv.bg (I.G.); kavradjieva@gmail.com (P.K.); 2 Department of Anaesthesiology Emergency and Intensive Care Medicine, Clinic of Anaesthesiology and Intensive Care, “St. George” University Hospital Plovdiv, Medical University of Plovdiv, 4002 Plovdiv, Bulgaria; ivaylo.minev@mu-plovdiv.bg; 3 Department of Endocrinology and Metabolic Diseases, Faculty of Medicine, “St. George” University Hospital Plovdiv, Medical University of Plovdiv, “Vassil Aprilov” 15A, 4000 Plovdiv, Bulgaria

**Keywords:** cleft palate, primary palatoplasty, oro-nasal fistula, intravelar palatoplasty, Veau–Wardill–Kilner technique, speech outcomes, secondary surgery

## Abstract

**Background**: Oro-nasal fistula formation remains one of the most common complications following primary palatoplasty, while the influence of surgical technique on fistula incidence and characteristics remains controversial. This study aimed to compare the occurrence and features of oro-nasal fistulas after two primary palatal repair techniques. **Methods**: A retrospective comparative analysis was conducted in patients undergoing one-stage primary palatoplasty using either closed intravelar palatoplasty or a modified Veau–Wardill–Kilner pushback technique. Oro-nasal fistulas were evaluated according to presence, size, anatomical location, and functional impact. Secondary corrective procedures, including fistula repair and pharyngoplasty, were also analyzed. **Results**: Oro-nasal fistula formation was significantly associated with the surgical technique. Closed intravelar palatoplasty demonstrated a significantly lower fistula rate compared with the modified Veau–Wardill–Kilner technique. Cleft type and syndromic status were not independently associated with fistula development. Rates of secondary corrective procedures and pharyngoplasty were significantly lower in the intravelar group. **Conclusions**: Surgical technique plays a decisive role in oro-nasal fistula development after primary palatoplasty. Muscle-oriented repair with limited incisions is associated with reduced fistula formation and a lower need for secondary surgical interventions.

## 1. Introduction

Cleft lip and palate are among the most common congenital craniofacial anomalies worldwide, with a reported global prevalence ranging between 1 in 700 and 1 in 1000 live births. These conditions represent a significant public health concern due to their impact on feeding, speech development, hearing, facial growth, and psychosocial well-being [1,2]. Genetic and environmental factors play a key role in the etiology of non-syndromic cleft lip and palate [3]. The primary objectives of palatal repair include separation of the oral and nasal cavities, restoration of normal palatal musculature, achievement of competent velopharyngeal function, and facilitation of normal speech development.

Despite advances in surgical techniques and perioperative care, oro-nasal fistula formation remains the most frequent postoperative complication following primary palatoplasty. Reported fistula rates vary widely in the literature, ranging from 0% to 60%, largely due to differences in surgical techniques, patient populations, cleft severity, and inconsistent definitions of fistula inclusion [4,5,6].

Multiple factors have been proposed as predictors of fistula formation, including cleft width, bilateral involvement, syndromic association, and surgical technique [4,5]. Among these variables, the impact of the operative method remains a subject of ongoing debate. Techniques involving extensive mucoperiosteal elevation and lateral relaxing incisions may increase tissue tension and compromise vascularity, thereby predisposing to fistula formation and impaired maxillary growth [5,7,8]. In contrast, muscle-oriented techniques with limited incisions aim to preserve blood supply, minimize scarring, and optimize velopharyngeal function.

Veau, Wardill, and Kilner originally described the classical principles of pushback palatoplasty. The Veau–Wardill–Kilner pushback palatoplasty remains widely used due to its ability to achieve palatal lengthening, particularly in wider clefts [9,10,11]. Closed intravelar palatoplasty emphasizes anatomical reconstruction of the levator veli palatini muscle and has been associated with minimal mucoperiosteal dissection, improved speech outcomes and reduced velopharyngeal insufficiency [12,13,14]. Sommerlad further refined this approach, emphasizing complete muscle dissection and functional repositioning [12]. Modified intravelar techniques with minimal incisions aim to preserve vascularity and reduce scar formation [15].

Although several studies have compared different palatoplasty techniques, direct comparisons between closed intravelar palatoplasty with minimal incisions and the modified Veau–Wardill–Kilner pushback technique remain limited. The present study provides a large single-center comparative analysis focusing not only on fistula incidence but also on anatomical localization, speech significance, and the need for secondary corrective procedures.

The present study aims to provide a comparative evaluation of oro-nasal fistulas occurrence, size and localization following these two operative techniques, and their association with secondary corrective procedures.

## 2. Materials and Methods

### 2.1. Study Design

A single-center, retrospective, comparative study was conducted to evaluate postoperative outcomes following primary palatoplasty performed using two different surgical techniques. The study included patients with unilateral, bilateral cleft lip and palate, as well as isolated cleft palate. The primary objective was to assess the incidence, size, localization, and clinical relevance of postoperative oro-nasal fistulas.

### 2.2. Study Population

Medical records of patients treated between 2005 and 2022 at the Department of Plastic, Reconstructive and Aesthetic Surgery for Children, University Hospital “St. George”, Plovdiv, Bulgaria, were retrospectively reviewed. Patients were included consecutively according to the surgical technique applied during the respective time periods.

### 2.3. Inclusion and Exclusion Criteria

Inclusion Criteria

- Patients with unilateral and/or bilateral cleft lip, alveolar ridge, and palate, or isolated cleft palate.
- Age between 4 and 15 years at the time of postoperative assessment.
- Availability of complete medical records.
- Signed written informed consent obtained from parents or legal guardians.
- Postoperative speech evaluation performed by a certified speech therapist.

The selected age range was chosen to allow for reliable long-term assessment of fistula occurrence and speech outcomes.

Exclusion Criteria

- Incomplete clinical documentation.
- Previous palatal surgery performed at another institution.

### 2.4. Electronic Medical Registry and Follow-Up

Clinical data were obtained from the Electronic Medical Register for Facial Anomalies (EMRFA), a national electronic database designed for longitudinal follow-up of patients with congenital facial anomalies [16]. The registry includes structured demographic, clinical, surgical, and functional outcome data and is routinely used for multidisciplinary care and outcome assessment.

Each patient has an individual electronic profile containing standardized information on diagnosis, operative interventions, postoperative outcomes, and adjunctive therapies. Data are entered prospectively by members of the multidisciplinary cleft team, including plastic surgeons, speech therapists, orthodontists, and other specialists. The registry allows for systematic upload of clinical documentation, including photographs, audio and video recordings, and structured assessment forms.

Patients can be grouped according to cleft type, age, sex, and surgical technique, enabling comparative statistical analyses. Speech evaluations, orthodontic assessments, and documentation of postoperative complications, including oro-nasal fistulas and secondary procedures, are recorded in predefined fields. Psychosocial parameters and global specialty-specific outcome ratings have also been integrated into the registry.

At the time of analysis, the EMRFA registry contained data on more than 1200 patients, with continuous enrollment and long-term follow-up from birth through adolescence. Data extracted for the present study were anonymized and used solely for research purposes.

### 2.5. Surgical Timing

In patients with cleft lip and palate, primary lip repair was performed between 1 and 3 months of age. Primary palatoplasty was carried out between 9 and 12 months of age in all patients.

### 2.6. Surgical Techniques

Patients were divided into two groups based on the surgical technique used for primary palatoplasty:

1. Closed intravelar palatoplasty with minimal incisions (*n* = 178).
2. Modified Veau–Wardill–Kilner pushback palatoplasty (*n* = 204).

Both techniques included intentional incision of the nasal mucosal layer to facilitate palatal elongation and optimize velopharyngeal function. The initial surgical intention was to perform closed intravelar palatoplasty whenever feasible. However, the final choice of technique was determined intraoperatively based on cleft morphology, tissue mobility, and the ability to achieve tension-free closure. When adequate closure could not be achieved with minimal tension, the procedure was converted to a modified Veau–Wardill–Kilner pushback palatoplasty. Therefore, the allocation of patients to the two surgical techniques was based on intraoperative assessment rather than preoperative selection.

The main technical differences between the two techniques are summarized to highlight factors influencing vascularity, tissue tension, and postoperative outcomes.

#### 2.6.1. Closed Intravelar Palatoplasty with Minimal Incisions

**Figure 1:**
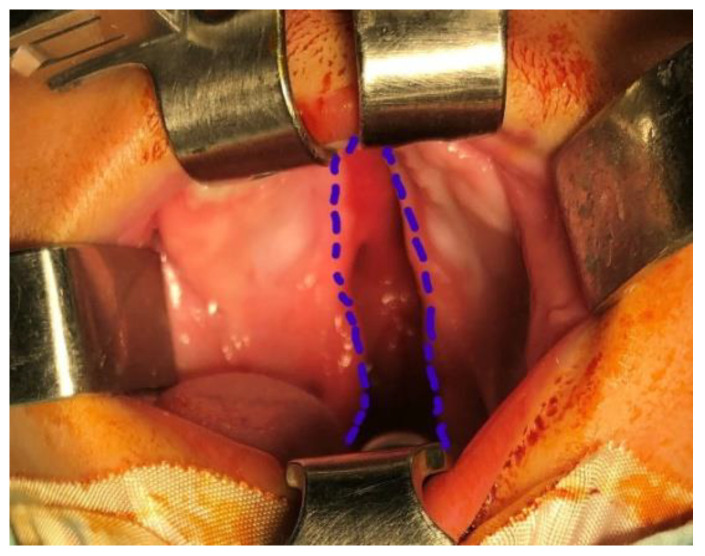
Incisions in the hard palate.

Closed intravelar palatoplasty was performed using minimal incisions without lateral relaxing cuts. Incisions were placed along the cleft margins, extending from the alveolar ridge to the base of the hemiuvulae. In the hard palate, dissection was performed at the junction between the oral mucosa and vomerine mucosa, while in the soft palate the incision was positioned slightly below the oral–nasal mucosal junction (Figure 1).

Bilateral mucoperiosteal flaps were elevated with careful identification and preservation of the greater palatine neurovascular bundles. The nasal mucosa of both the hard and soft palate was meticulously dissected from the underlying structures and closed in the midline using absorbable sutures, without the use of a vomer flap.

**Figure 2:**
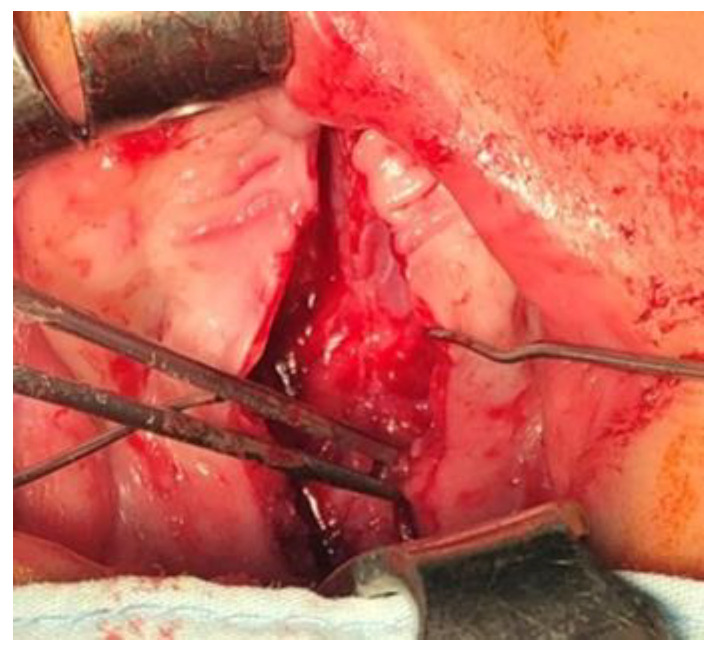
Identification and preservation of a. palatina major.

**Figure 3:**
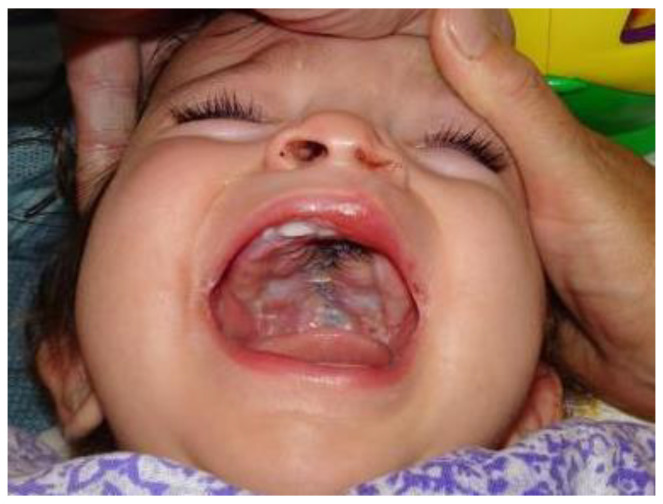
Intravelar palatoplasty performed without lateral releasing incisions.

A key step of this modified technique is the transverse incision of the reconstructed nasal layer at the junction of the hard and soft palate. This maneuver allows for intraoperative elongation of the soft palate (Figure 2). The levator veli palatini muscles were completely released from their abnormal insertions, transversely repositioned, and sutured to recreate a functional muscular sling. An internal fracture of the hamulus was performed to reduce tension and facilitate muscle mobilization, without employing Limberg osteotomy. Oral mucosal closure was achieved in the midline without tension, and the uvula was reconstructed in layers to obtain a symmetrical and anatomically functional velum (Figure 3).

#### 2.6.2. Modified Veau–Wardill–Kilner Palatoplasty

**Figure 4:**
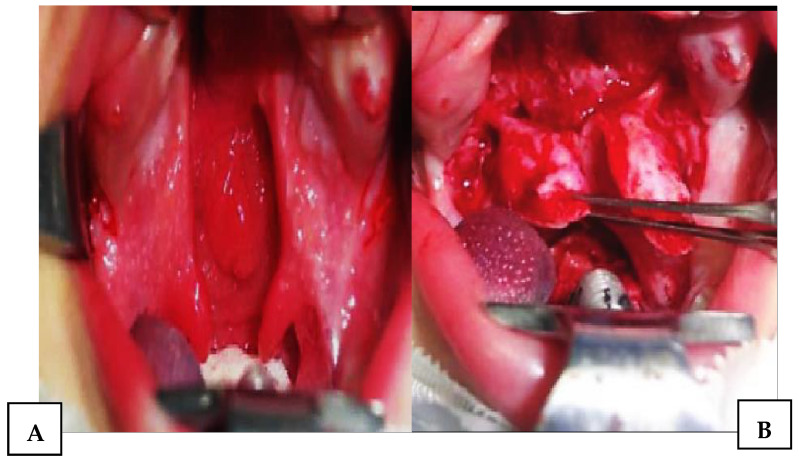
(**A**). Incisions along the cleft margins with anterior and lateral relaxing incisions. (**B**) The formation of triangular mucoperiosteal flaps based on the posterior branch of the a. palatina major.

**Figure 5:**
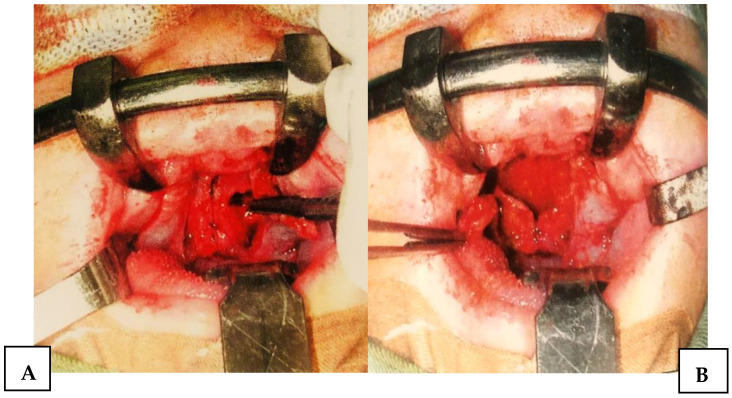
(**A**) Transverse incision of the nasal layer. (**B**) Elongation of the nasal layer base and palatal muscles.

**Figure 6:**
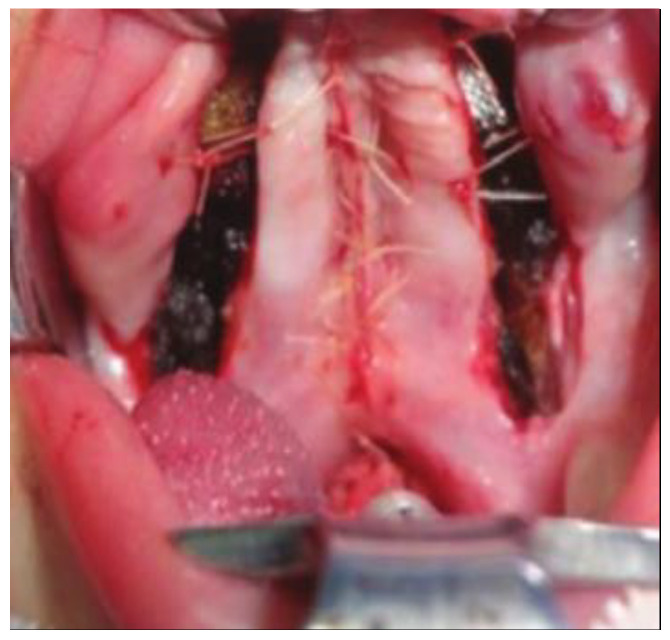
Midline closure of the two mucoperiosteal flaps leaving extensive anterior and lateral areas of exposed maxillary bone along the alveolar ridge.

After standard surgical exposure, incisions were made along the cleft margins in combination with anterior and lateral relaxing incisions. This approach allowed for elevation of two unipediculated triangular mucoperiosteal flaps based on the posterior bundle of the greater palatine artery (Figure 4). In contrast to the original Veau–Wardill–Kilner technique, our modification included transverse incision of the reconstructed nasal mucosal layer, enabling additional intraoperative palatal lengthening. No vomer flap was used for nasal layer reconstruction. The mucoperiosteal flaps were transposed in a V–Y fashion to achieve elongation of the hard and soft palate, and the levator veli palatini muscles were repositioned toward the posterior pharyngeal wall (Figure 5 and Figure 6). This technique resulted in extensive anterior and lateral areas of exposed maxillary bone, which healed by secondary intention and were associated with increased scar formation and potential restriction of midfacial growth.

All patients were treated in the national referral cleft center, which provides centralized multidisciplinary care for cleft patients in the country. All procedures were performed by two experienced cleft surgeons working within the same team and following standardized institutional protocols. Given the long-standing experience of the surgeons and the consistent operative approach used during the study period, a significant learning curve effect is unlikely to have influenced the results.

### 2.7. Outcome Measures

Postoperative outcomes were assessed during long-term follow-up. In our cleft center, all patients are routinely followed from birth until completion of craniofacial growth. Comprehensive clinical assessments are performed every two years and include evaluation of palatal integrity, fistula occurrence, and functional outcomes.

The following postoperative parameters were evaluated:

- Presence or absence of oro-nasal fistula.
- Fistula size (small, clinically insignificant; large, clinically significant; or wound dehiscence).
- Anatomical localization (anterior oro-nasal, hard–soft palate junction, soft palate).
- Impact on speech, defined by the presence of hypernasality.
- Requirement for secondary corrective surgery, including reoperative palatoplasty or pharyngoplasty.

Oro-nasal fistulas were classified according to their size, anatomical location, and clinical relevance based on predefined criteria.

### 2.8. Speech Evaluation

Speech outcomes were assessed as part of the routine postoperative follow-up using standardized perceptual speech assessment protocols. Evaluations were performed by two experienced speech therapists specialized in the management of patients with cleft lip and palate. Speech assessment was not blinded to the surgical technique used.

Speech evaluation was conducted to enable comparative analysis of functional outcomes between the two operative techniques—closed intravelar palatoplasty with minimal incisions and the Modified Veau–Wardill–Kilner technique.

The assessment protocol was based on the Eurocleft perceptual speech evaluation scale, adapted to the Bulgarian phonetic system. This adaptation allows for a reliable evaluation of speech characteristics specific to the phonological and articulatory features of the Bulgarian language.

Speech data collection followed a standardized protocol and included the following:

- Audio and/or video recordings of speech samples. Patients were required to pronounce age-appropriate words and sentences of increasing complexity. In addition, a standardized text containing all Bulgarian consonants and vowels was used in children older than 5 years.
- Perceptual evaluation of resonance, nasal emission, and nasal turbulence using a five-point ordinal scale according to the adapted Eurocleft protocol.
- Assessment of articulation during repetition of isolated sounds, syllables, words, and complete sentences.
- Evaluation of speech intelligibility during spontaneous speech using a five-point scale based on the Eurocleft classification.

Speech evaluation was performed during long-term follow-up visits when patients were between 4 and 15 years of age. The age of 4–6 years is considered the most reliable period for comprehensive speech assessment in cleft palate patients, as speech production becomes sufficiently stable and cooperative testing is possible. Older patients were also included in order to evaluate long-term functional outcomes.

Fistulas were classified as speech-significant when associated with clinically evident hypernasality and impaired speech intelligibility.

Inter-rater reliability between the two speech therapists was assessed using Cohen’s kappa coefficient. The obtained value (κ > 0.80) indicated very good agreement and high reliability of the speech evaluation.

Speech therapy represents an essential component of the multidisciplinary management of children with cleft lip and palate. Given the long-term nature of speech rehabilitation and the need for consistent daily practice, standardized diagnostic and assessment criteria are crucial for reliable outcome comparison.

For the purposes of this study, speech-related data were extracted from the institutional Electronic Medical Record system for patients with facial anomalies. The analyzed parameters included key speech indicators directly affecting intelligibility, such as resonance, nasal emission, facial grimacing, voice quality, speech development, vowel production, consonant articulation, and documented recommendations for speech therapy.

### 2.9. Statistical Analysis

Statistical analysis was performed using SPSS software (version 26.0; IBM Corp., Armonk, NY, USA). Continuous variables were expressed as the mean ± standard deviation, while categorical variables were presented as frequencies and percentages. Group comparisons were conducted using chi-square tests, Z-tests. Correlation analyses and logistic regression models were applied to identify predictors of oro-nasal fistula formation. A *p*-value < 0.05 was considered statistically significant.

### 2.10. Ethical Considerations

The study was approved by the institutional ethics committee of University Hospital “St. George”, Plovdiv. All procedures were conducted in accordance with the Declaration of Helsinki, and written informed consent was obtained from all patients’ parents or legal guardians.

## 3. Results

### 3.1. Patient Characteristics

A total of 382 patients were included in the analysis and divided into two groups according to the surgical technique used for primary palatoplasty. Of these, 204 patients (53.4%) underwent modified Veau–Wardill–Kilner palatoplasty, while 178 patients (46.6%) were treated using closed intravelar palatoplasty with minimal incisions.

The overall mean age at the time of postoperative assessment was 9.8 years (range: 4–15 years). Patients treated with intravelar palatoplasty had a significantly lower mean age at assessment compared with those treated using the Veau–Wardill–Kilner technique (8.27 vs. 11.21 years, *p* < 0.05).

The study population included 210 males (55.0%) and 172 females (45.0%), with no statistically significant difference in sex distribution between the two surgical groups (χ^2^ = 1.46, *p* > 0.05).

Syndromic clefts were identified in 9.7% of the overall cohort. The proportion of patients with associated genetic syndromes was significantly higher in the intravelar palatoplasty group compared with the Modified Veau–Wardill–Kilner group (χ^2^ = 5.49, *p* = 0.019). The following table presents the comparative demographic and clinical characteristics of patients in both groups (Table 1).

### 3.2. Distribution of Cleft Types

**Figure 7:**
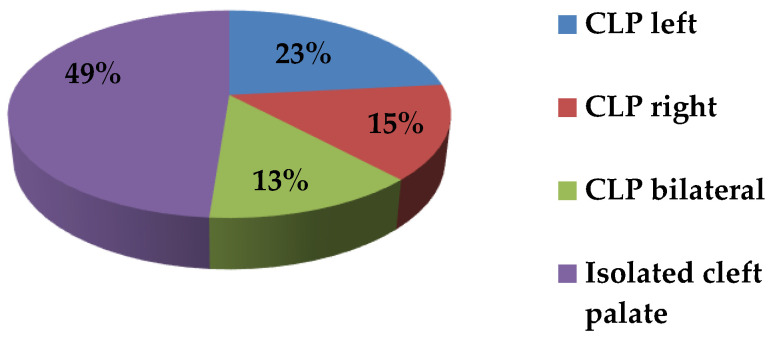
Distribution of patients according to cleft type.

Among all patients, 186 (48.8%) presented with isolated cleft palate, 90 (23.4%) with left-sided unilateral cleft lip, alveolar ridge, and palate, 56 (14.7%) with right-sided unilateral cleft, and 50 (13.1%) with bilateral cleft lip and palate (Figure 7).

The baseline distribution of cleft types differed significantly between the two surgical techniques (Table 2). Isolated cleft palate was more frequently treated using intravelar palatoplasty, whereas unilateral and bilateral cleft lip and palate were more commonly managed with the modified Veau–Wardill–Kilner technique (Z-test, *p* < 0.05).

### 3.3. Incidence of Oro-Nasal Fistulas

Postoperative oro-nasal fistulas were identified in 110 of 382 patients (28.8%). The incidence of fistula formation differed significantly between the two surgical techniques.

**Figure 8:**
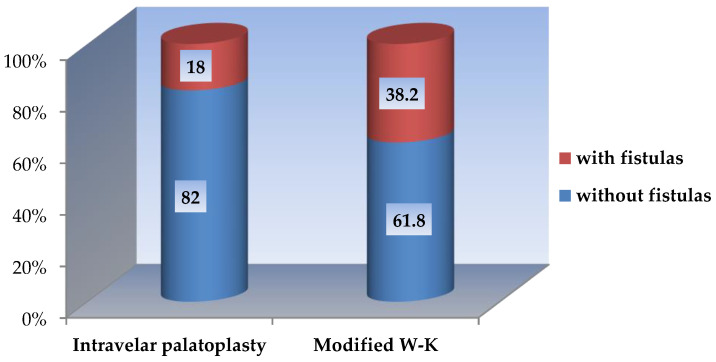
Incidence of postoperative oro-nasal fistulas according to surgical technique.

In the intravelar palatoplasty group, fistulas were observed in 32 patients (18.0%), whereas in the modified Veau–Wardill–Kilner group fistulas occurred in 78 patients (38.2%). The difference in fistula incidence between the two groups was statistically significant (Z-test, *p* < 0.05) (Figure 8).

**Figure 9:**
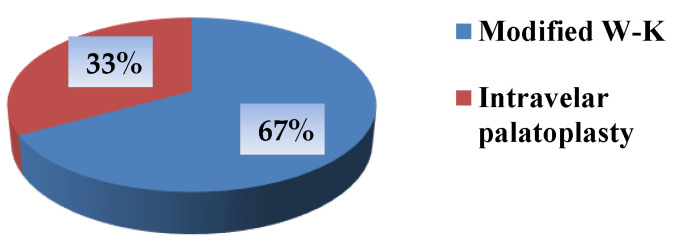
Distribution of fistulas by two operative methods.

Patients who developed postoperative fistulas after palatoplasty using the modified Veau–Wardill–Kilner technique accounted for 67% (*n* = 78) of all patients with fistulas. In contrast, patients operated on using the closed intravelar palatoplasty with minimal incisions represented 33% (*n* = 32) of the total number of patients with fistulas. This difference was statistically significant (*p* < 0.05) (Figure 9).

### 3.4. Fistula Size and Localization

**Figure 10:**
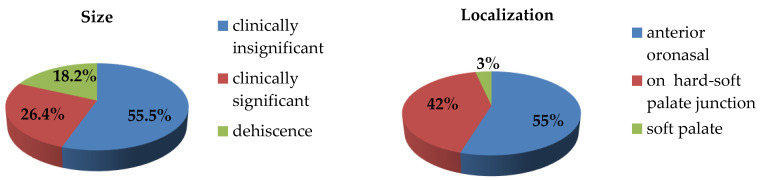
Localization and size of fistulas in all operated patients.

Fistulas were categorized as clinically insignificant (1–2 mm), clinically significant (>2 mm), or wound dehiscence. Of the 110 identified fistulas, 61 (55.5%) were classified as small and clinically insignificant, 29 (26.4%) as large and clinically significant, and 20 (18.2%) as wound dehiscence (Figure 10).

No association was found between fistula size and the surgical technique used. In both groups, clinically insignificant fistulas predominated, accounting for 53.1% of fistulas in the intravelar palatoplasty group and 56.4% in the Modified Veau–Wardill–Kilner group. Clinically significant fistulas across all forms of cleft lip and palate represented 28.1% in the intravelar group and 25.6% in the Modified Veau–Wardill–Kilner group.

**Figure 11:**
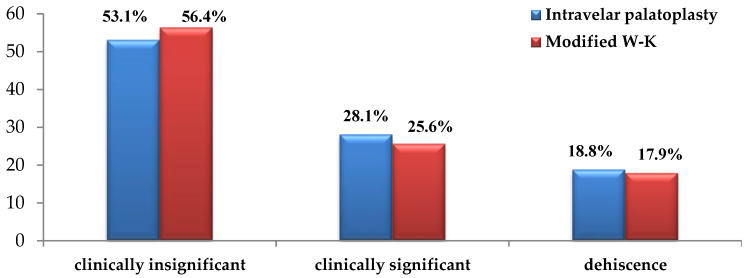
Distribution of fistulas according to size in two patient groups.

Cases of wound dehiscence were also comparable between groups, occurring in 18.8% of patients treated with the minimal-incision intravelar technique and in 17.9% of those treated with the modified Veau–Wardill–Kilner technique. The differences between the two groups did not reach statistical significance (χ^2^ = 0.105; *p* > 0.05) (Figure 11).

**Figure 12:**
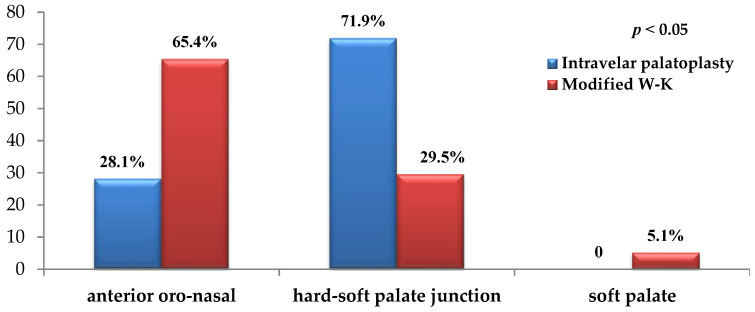
Distribution of fistulas according to localization.

According to anatomical region, fistulas were classified as anterior oro-nasal, located at the hard–soft palate junction, or isolated to the soft palate. Regarding anatomical localization, fistulas in the intravelar palatoplasty group most frequently occurred at the junction of the hard and soft palate, whereas anterior oro-nasal fistulas predominated in the modified Veau–Wardill–Kilner group. A statistically significant association was observed between surgical technique and fistula localization (*p* < 0.05) (Figure 12).

### 3.5. Speech-Relevant Fistulas

**Figure 13:**
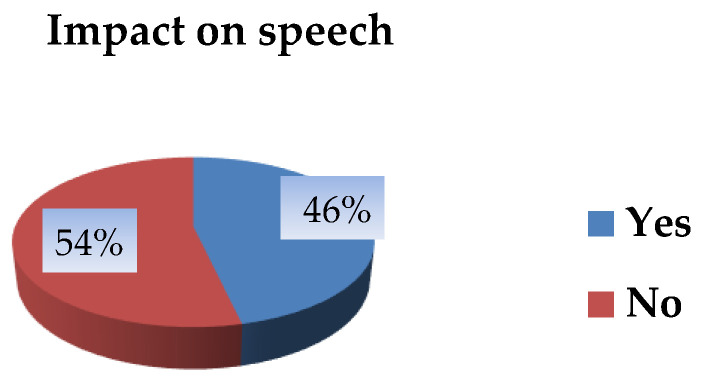
Distribution of postoperative fistulas according to impact on speech.

Of all postoperative fistulas identified in patients treated with both surgical techniques, 51 out of 110 (46.4%) were classified as speech-significant, as they were associated with clinically evident hypernasality. In the remaining 59 cases (53.6%), no impact on speech was observed (Figure 13).

**Figure 14:**
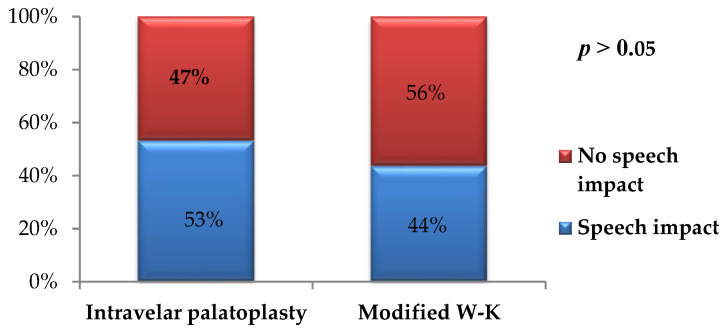
Distribution of oro-nasal fistulas according to impact on speech in both surgical groups.

In the intravelar palatoplasty group, 17 of 32 postoperative fistulas (53.1%) were speech-significant, whereas 15 (46.9%) did not affect speech. In contrast, in the modified Veau–Wardill–Kilner group, 34 of 78 fistulas (43.6%) were classified as speech-significant. When analyzed relative to the total number of postoperative fistulas within each surgical technique, the difference in the proportion of speech-significant fistulas between the two groups was not statistically significant (*p* > 0.05) (Figure 14).

### 3.6. Reoperations and Pharyngoplasty

**Figure 15:**
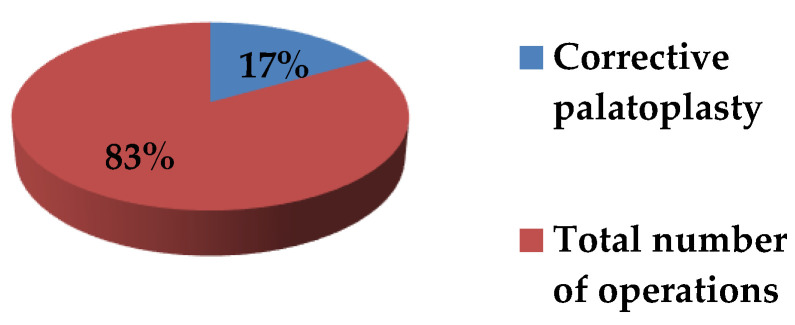
Rate of secondary corrective palatoplasty.

From a total of 382 patients included in the study, 64 (16.8%) required secondary corrective palatoplasty (Figure 15).

**Figure 16:**
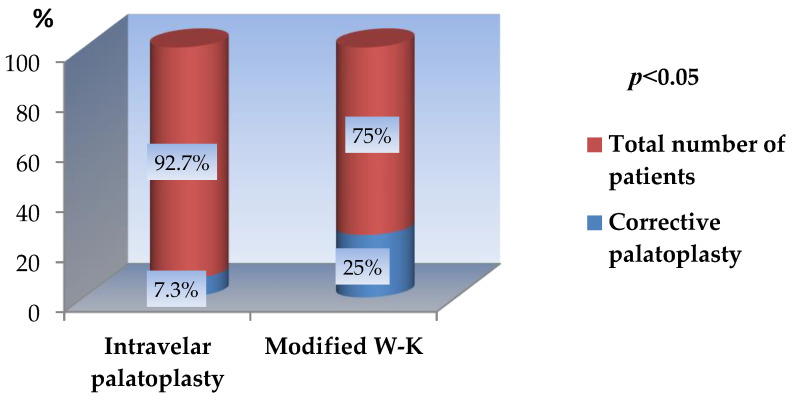
Corrective palatoplasty in both groups.

A statistically significant association was identified between the primary surgical technique and the need for secondary corrective procedures. Patients treated with closed intravelar palatoplasty demonstrated a significantly lower rate of reoperative palatoplasty for fistula closure compared with those treated using the modified Veau–Wardill–Kilner technique. Specifically, the reoperation rate was 7.3% in the intravelar palatoplasty group versus 25.0% in the Modified Veau–Wardill–Kilner group, a difference that reached statistical significance (*p* < 0.05) (Figure 16).

Out of the total 382 patients treated at our center, 31 (8.1%) underwent secondary pharyngoplasty.

A similar association was observed with respect to secondary pharyngoplasty. The procedure was performed significantly more frequently in patients treated with the modified Veau–Wardill–Kilner technique, reflecting the higher incidence of clinically relevant fistulas and velopharyngeal dysfunction in this group.

**Figure 17:**
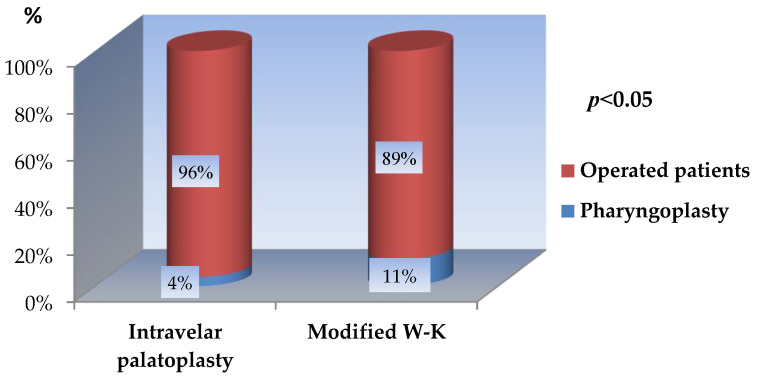
Distribution of patients requiring pharyngoplasty according to surgical technique.

Among the 178 patients who underwent closed intravelar palatoplasty with minimal incisions, 7 (3.9%) required secondary pharyngoplasty, compared with 23 of 204 patients (11.3%) treated using the modified Veau–Wardill–Kilner technique. The difference between the two groups was statistically significant (Z-test, *p* < 0.05) (Figure 17).

### 3.7. Correlation and Regression Analyses: Predictors of Fistula Formation

Correlation analysis demonstrated a weak positive association between fistula formation and surgical technique (r = 0.28). A weak correlation was also observed between surgical technique and speech-significant fistulas (r = 0.04).

A multivariable logistic regression analysis was performed to assess the effect of surgical technique, cleft type, and presence of an associated syndrome on the probability of postoperative oro-nasal fistula formation (Table 3). The regression model was statistically significant (χ^2^(4) = 38.97, *p* < 0.001) and explained 13.0% of the variance in fistula occurrence (Nagelkerke R^2^). The model correctly classified 71.0% of cases.

The surgical technique emerged as the only statistically significant predictor of fistula formation. Use of minimal-incision intravelar palatoplasty was associated with a significantly reduced likelihood of fistula occurrence compared with the Modified Veau–Wardill–Kilner technique (B = −0.539, *p* = 0.047), corresponding to an odds ratio of 0.58 (95% CI: 0.34–0.99). Neither cleft type (overall Wald = 0.917, *p* = 0.821) nor presence of an associated syndrome (OR = 1.43, *p* = 0.439) showed a statistically significant association with fistula occurrence.

Logistic regression analysis identified the surgical technique as a significant predictor of fistula formation. Closed intravelar palatoplasty was associated with a reduced probability of fistula occurrence, while cleft type and syndromic status were not significant predictors.

## 4. Discussion

The management of cleft palate remains one of the most challenging areas in reconstructive surgery, with the primary goals of achieving effective velopharyngeal competence, minimizing postoperative complications, and preserving midfacial growth. Oro-nasal fistula formation continues to be the most common and clinically relevant complication following primary palatoplasty, directly affecting speech outcomes and frequently necessitating secondary surgical intervention. Despite numerous refinements in surgical techniques, reported fistula rates remain highly variable, reflecting differences in patient selection, cleft severity, operative methods, and definitions of fistula across studies.

In the present study, we performed a comprehensive comparative analysis of two widely used surgical techniques for primary palatal repair—closed intravelar palatoplasty with minimal incisions and a modified Veau–Wardill–Kilner pushback technique—in a large, single-center cohort with long-term follow-up. Our findings demonstrate a significantly lower overall incidence of postoperative oro-nasal fistulas in patients treated with intravelar palatoplasty compared with those undergoing the modified Veau–Wardill–Kilner technique. Importantly, this difference remained evident when only clinically significant fistulas and wound dehiscence were considered, underscoring the clinical relevance of the observed reduction. Although both techniques involved transversal incision of the nasal mucosal layer to achieve palatal elongation, the intravelar approach preserved collateral blood supply and minimized lateral incisions, potentially explaining the reduced fistula rate and lower need for secondary surgery. The reduced rates of reoperation and pharyngoplasty in the intravelar group further support the functional advantages of muscle-oriented palatal reconstruction with minimal tissue trauma.

### 4.1. Surgical Technique and Fistula Formation

Oro-nasal fistula remains the most common complication following primary palatoplasty [4,5,6]. Numerous classification systems have been proposed based on fistula size and anatomical localization [17,18]. In the present study, fistulas were categorized according to size as small (clinically insignificant), large (clinically significant), or wound dehiscence. Small fistulas were defined as measuring 1–2 mm, whereas large fistulas exceeded 2 mm in diameter. Wound dehiscence referred to cases of complete breakdown of the operative suture line.

From an anatomical perspective, fistulas may be classified as prealveolar, alveolar, or postalveolar. The Pittsburgh classification system further subdivides fistulas into seven types based on precise anatomical location [17,18]. Due to limitations inherent in retrospective record documentation, it was not feasible to classify all fistulas according to the full Pittsburgh system. Therefore, anatomical localization was simplified into three clinically relevant categories: anterior oronasal fistulas, fistulas at the junction of the hard and soft palate, and fistulas confined to the soft palate.

The association between surgical technique and fistula formation observed in our study is consistent with previous reports emphasizing the critical role of tissue handling, preservation of vascular supply, and tension-free closure in palatal repair [4,5,19,20,21]. Techniques involving extensive mucoperiosteal elevation and lateral relaxing incisions—such as the modified Veau–Wardill–Kilner pushback procedure—have been associated with higher fistula rates in multiple studies [4,6,19]. The presence of large areas of exposed maxillary bone healing by secondary intention may promote scar contraction, local ischemia, and wound breakdown, thereby increasing the risk of fistula development [7,8,22].

In contrast, closed intravelar palatoplasty with minimal incisions is designed to preserve palatal vascularity and minimize surgical trauma [12,15]. In our cohort, this approach was associated with a significantly lower incidence of fistula formation, despite the intentional transverse incision of the nasal mucosal layer employed in both techniques. These findings suggest that limitation of lateral and anterior incisions—and the consequent reduction in exposed bone surface—may be more critical for fistula prevention than preservation of the nasal mucosa alone.

### 4.2. Localization and Clinical Significance of Fistulas

We observed a distinct pattern of fistula localization between the two surgical groups. Fistulas following intravelar palatoplasty predominantly occurred at the junction of the hard and soft palate, whereas anterior oro-nasal fistulas were more common after the modified Veau–Wardill–Kilner procedure. This distribution likely reflects both the surgical design and the underlying cleft morphology, as wider clefts were more frequently managed with the pushback technique in our institution.

Although no significant association was found between fistula size and surgical technique, the overall proportion of clinically insignificant fistulas was higher in both groups. Nevertheless, patients treated with the modified Veau–Wardill–Kilner technique demonstrated a significantly higher rate of speech-significant fistulas when analyzed in relation to the total number of operated patients. This finding highlights that fistula incidence alone may not fully capture the functional impact of surgical outcomes and emphasizes the importance of speech-related evaluation in postoperative assessment.

### 4.3. Speech Outcomes and Secondary Procedures

Speech outcomes represent a critical endpoint in cleft palate surgery [23,24]. Velopharyngeal dysfunction and hypernasality are closely associated with both fistula formation and inadequate muscle reconstruction [14,23]. Previous systematic analyses have demonstrated variable fistula incidence across palatoplasty techniques [25]. The presence of postpalatoplasty fistulas has important functional implications, as higher fistula rates have been associated with an increased need for secondary speech surgery [24,26,27,28]. In this context, surgical techniques that reduce fistula incidence may contribute not only to improved wound healing but also to better long-term speech outcomes and a reduced need for secondary interventions.

Nearly half of all fistulas identified in our cohort were associated with clinically evident hypernasality. While the proportion of speech-significant fistulas among patients with fistulas did not differ significantly between techniques, the overall burden of speech-related complications was greater in the modified Veau–Wardill–Kilner group due to the higher absolute fistula rate.

Recent studies continue to support tissue-preserving, muscle-oriented palatoplasty techniques as a means of reducing complications and improving functional outcomes [19,20,21,22,25,28,29,30,31,32,33,34,35].

Secondary corrective procedures, including reoperative palatoplasty and pharyngoplasty, are frequently required in patients with clinically significant fistulas or velopharyngeal insufficiency [26,27]. Consistent with these findings, secondary corrective procedures were performed significantly more often in patients treated with the modified Veau–Wardill–Kilner technique. However, the higher rate of secondary procedures in this group may partly reflect the greater proportion of more complex cleft types treated with this technique. Nevertheless, this association reinforces the clinical importance of fistula prevention, as secondary surgeries increase patient morbidity, healthcare costs, and psychosocial burden.

### 4.4. Role of Nasal Layer Incision and Palatal Lengthening

A distinctive aspect of our surgical protocol is the intentional transverse incision of the reconstructed nasal mucosal layer in both techniques. Although this maneuver introduces a potential risk for fistula formation, it enables effective intraoperative elongation of the soft palate and facilitates restoration of a functional muscular sling [12,13,14,23]. Previous studies have demonstrated that palatal lengthening is associated with improved velopharyngeal function and reduced rates of velopharyngeal insufficiency [13,24,27]. Our results suggest that the benefits of nasal layer incision in achieving adequate palatal length and muscle repositioning may outweigh the associated risks, particularly when combined with a tissue-sparing approach such as intravelar palatoplasty [36]. The significantly lower rates of pharyngoplasty observed in this group further support the functional advantages of this strategy [37,38].

### 4.5. Regression Analysis and Independent Predictors

Multivariable logistic regression analysis demonstrated that operative technique was an independent predictor of postoperative fistula formation. After adjustment for cleft type and syndromic status, intravelar palatoplasty with minimal incisions was associated with a significantly lower probability of fistula occurrence compared with the modified Veau–Wardill–Kilner technique. Neither cleft type nor the presence of a syndrome independently influenced fistula development. Logistic regression analysis was performed according to established statistical methodology [39,40].

### 4.6. Clinical Implications

Our findings provide clinically meaningful evidence that surgical technique plays a pivotal role in postoperative outcomes following primary palatoplasty. Closed intravelar palatoplasty with minimal incisions appears to offer a favorable balance between effective palatal lengthening, reduced fistula formation, and improved functional outcomes.

### 4.7. Limitations

Several limitations of this study should be acknowledged. First, the retrospective design inherently limits the ability to control for all potential confounding variables. Second, the distribution of cleft types differed between the two surgical groups, with isolated cleft palate being more common in the intravelar palatoplasty group and unilateral or bilateral cleft lip and palate more frequently treated with the modified Veau–Wardill–Kilner technique. This difference reflects the historical evolution of surgical practice in our center and the gradual introduction of muscle-oriented palatal repair. As a result, patients treated with intravelar palatoplasty were generally operated on in more recent years and had a lower mean age at assessment. In addition, because the study spans a long retrospective period and procedures were performed by two surgeons within the same surgical team, a formal analysis of a potential learning curve effect could not be performed. These factors should therefore be considered when interpreting the comparative outcomes of the two surgical techniques.

## 5. Conclusions

Within the limitations of this single-center retrospective study, surgical technique was associated with differences in postoperative outcomes following primary palatoplasty. Closed intravelar palatoplasty with minimal incisions showed a lower incidence of oro-nasal fistulas and fewer secondary corrective procedures compared with the modified Veau–Wardill–Kilner technique. Preservation of palatal vascularity and minimization of lateral and anterior incisions appear critical for fistula prevention. Differences in cleft type distribution between groups should be considered when interpreting these results.

## Figures and Tables

**Table 1:** Demographic and clinical characteristics of patients according to surgical technique.

Surgical Technique/ Index	Intravelar Palatoplasty Nr/%	Modified W-K * Nr/%	*p*
Patients	178/100	204/100	
Age			
Mean ± SE	8.27 ± 0.2	11.21 ± 0.2	*p* < 0.05
Range	4–15	4–15	
Sex			
Male	92/51.7	118/57.8	*p* > 0.05
Female	86/48.3	86/42.2	
Syndromic	24/13.5	13/6.4	
Non-syndromic	154/86.5	191/93.6	*p* < 0.05

* Modified Wardill–Kilner technique.

**Table 2:** Baseline distribution of cleft types according to surgical technique.

Surgical Technique/ CLP **	Intravelar Palatoplasty Nr/%	Modified W-K * Nr/%	*p*	Total Nr/%
Unilateral CLP	42/23.6	104/50.8	*p* < 0.05	146/38.1
Bilateral CLP	10/5.6	40/19.7	*p* < 0.05	50/13.1
Isolated CP ***	126/70.8	60/29.6	*p* < 0.05	186/48.8

* Modified Wardill–Kilner technique, ** Cleft lip and palate, *** Cleft palate.

**Table 3:** Logistic regression model to estimate probability of fistula occurrence with following variables: method, cleft type, and syndrome.

Variables	B	S.E.	Wald	df	Sig.	Exp(B)	95% C.I. for EXP(B)	
							Lower	Upper
Method (1)	−0.539	0.272	3.939	1	0.047	0.583	0.342	0.993
Cleft type			0.917	3	0.821			
Cleft type (1)	0.140	0.310	0.204	1	0.651	1.150	0.627	2.110
Cleft type (2)	0.337	0.353	0.910	1	0.340	1.401	0.701	2.799
Cleft type (3)	0.136	0.372	0.134	1	0.714	1.146	0.553	2.378
Syndrome (1)	0.355	0.459	0.598	1	0.439	1.426	0.580	3.508
Constant	−0.756	0.521	2.104	1	0.147	0.470		

Variables: method, cleft type, syndrome.

## Data Availability

The datasets generated and analyzed during the current study are not publicly available due to patient confidentiality and institutional data protection policies but are available from the corresponding author on reasonable request.
